# Traditional Chinese Medicine for Refractory Nephrotic Syndrome: Strategies and Promising Treatments

**DOI:** 10.1155/2018/8746349

**Published:** 2018-01-04

**Authors:** Xiao-Qin Wang, Lan Wang, Yuan-Chao Tu, Yuan Clare Zhang

**Affiliations:** ^1^Department of Chinese Medicine Nephrology, Hubei Provincial Hospital of TCM, Hubei University of Chinese Medicine, Wuhan, Hubei, China; ^2^Institute of Chinese Medicine Nephrology, Hubei Province Academy of Traditional Chinese Medicine, Wuhan, Hubei, China; ^3^Y. Clare Zhang Practice of Oriental Medicine, Tucson, AZ, USA

## Abstract

Refractory nephrotic syndrome (RNS) is an immune-related kidney disease with poor clinical outcomes. Standard treatments include corticosteroids as the initial therapy and other immunosuppressants as second-line options. A substantial proportion of patients with RNS are resistant to or dependent on immunosuppressive drugs and often experience unremitting edema and proteinuria, cycles of remission and relapse, and/or serious adverse events due to long-term immunosuppression. Traditional Chinese medicine has a long history of treating complicated kidney diseases and holds great potential for providing effective treatments for RNS. This review describes the Chinese medical theories relating to the pathogenesis of RNS and discusses the strategies and treatment options using Chinese herbal medicine. Available preclinical and clinical evidence strongly supports the integration of traditional Chinese medicine and Western medicine for improving the outcome of RNS. Herbal medicine such as* Astragalus membranaceus, Stephania tetrandra *S. Moore, and* Tripterygium wilfordii* Hook F can serve as the alternative therapy when patients fail to respond to immunosuppression or as the complementary therapy to improve therapeutic efficacy and reduce side effects of immunosuppressive agents. Wuzhi capsules (*Schisandra sphenanthera* extract) with tacrolimus and tetrandrine with corticosteroids are two herb-drug combinations that have shown great promise and warrant further studies.

## 1. Refractory Nephrotic Syndrome—A Rare Immune Disease with Serious Consequences

Nephrotic syndrome is a rare but serious kidney disease that affects children and adults worldwide. Its clinical presentations include peripheral edema, heavy proteinuria, and hypoalbuminemia, often with hyperlipidemia. The reported annual incidence is 2–7 per 100,000 children and 3 per 100,000 adults [1, 2]. Although occurring at a low rate, it is responsible for approximately 12% of all causes of end stage renal disease (ESRD) and up to 20% of ESRD in children [3]. The etiology of nephrotic syndrome ranges from primary glomerulonephritis to secondary diseases associated with drugs, infections, and neoplasia. The cause of primary nephrotic syndrome is complex and not well understood; however ample evidence indicates that it is an immune-mediated disorder leading to glomerular podocyte injury and increased glomerular permeability [4].

Primary nephrotic syndrome consists of three major pathophysiological subtypes—idiopathic membranous nephropathy (IMN), minimal change disease (MCD), and focal segmental glomerulosclerosis (FSGS). Their pathogenic mechanisms, albeit different in aspects, all involve immune damage of glomerular podocytes with low inflammatory nature.

IMN is generally considered an autoimmune disease, characterized by immune complex deposition and complement activation in the subepithelial space between glomerular podocytes and the glomerular basement membrane, which together contribute to functional disruption of the glomerular capillary wall [5]. The identification of two important podocyte autoantigens—secretory phospholipase A2 receptor (PLA2R1) and thrombospondin type 1 domain containing 7A protein (THSD7A)—and detection of their autoantibodies in 60–80% and 5–10% of patients with membranous nephropathy, respectively [6, 7], were regarded as landmark discoveries in understanding the molecular pathomechanism of IMN.

MCD and FSGS were traditionally described as separate entities. However, convincing evidence now suggests that they are in fact different manifestations of the same progressive disease, with FSGS being the more advanced stage than MCD [8]. Their immune pathogenesis is considered to arise from a systemic disturbance of T-cell function leading to the production of cytokines or other circulating permeability factors that cause direct or indirect impairment of glomerular function [9].

IMN is the leading cause of nephrotic syndrome in adults, but it is rare in children [1]. MCD and FSGS are the most common causes of childhood nephrotic syndrome. In adults, they each account for 10–15% and 40% of nephrotic syndrome cases, respectively [10].

The standard first-line treatment for nephrotic syndrome is corticosteroids. Although most children with nephrotic syndrome are sensitive to steroids, approximately 20% of children are steroid-resistant. Moreover, 80–90% of pediatric patients who respond initially to steroids experience relapse, and many develop steroid dependency following frequent relapses and repeated courses of steroid administration [11]. Refractory nephrotic syndrome (RNS) thus refers to the subset of patients with nephrotic syndrome who are steroid-resistant, or steroid-dependent, and experience frequent relapses [12]. RNS comprises 25–40% of nephrotic syndrome cases in children and adolescents [13], and its incidence is even greater (up to 70%) in adults [10]. For instance, MCD has over 50% relapse rate in adults [10], and 10–20% of the adult patients with MCD are steroid-resistant [14]. Due to the high incidence of RNS in adult nephrotic syndrome, the terms of nephrotic syndrome and RNS are often used interchangeably in medical practice and literature.

In recent years, second-line immunosuppressive therapies including cytotoxic agents, calcineurin inhibitors, mycophenolate mofetil, and rituximab have been widely used in RNS treatment with promising results. However, long-term use of steroids and some of these immunosuppressants causes serious adverse effects such as nephrotoxicity, hyperglycemia, dyslipidemia, osteoporosis, hypoimmunity, and high risk of infection. Moreover, the risk/benefit profile of these treatments is poor. Most patients with RNS still present severe nonremitting edema, heavy proteinuria, hypoalbuminemia, and sometimes decreased kidney function.

The limited success of currently available treatments for RNS has prompted active research into safer and more effective alternative therapies. Traditional Chinese medicine (TCM) has a long history of treating symptoms of kidney diseases such as edema and proteinuria. Through persistent trial and error, theorization, and retheorization, TCM has developed unique perspectives to explain nephrotic syndrome and accumulated rich clinical experience in treating this disease. Therefore, it makes sense to explore TCM for better RNS treatment.

In this article, we briefly review the current status of RNS treatment by conventional medicine. We describe the TCM theory for understanding the etiology and pathogenesis of RNS. We also discuss the TCM strategies and treatment options as alternative therapies or in a supportive role to improve the efficacy and reduce side effects of immunosuppressive drugs. Finally, we present two herb-drug combinations with promising clinical results and discuss possible mechanisms of action in the treatment of RNS.

## 2. Current Status of RNS Treatment

For over 40 years, empirical treatment with immunosuppressive agents including corticosteroids has been the mainstay of therapy for nephrotic syndrome [15]. This is largely due to insufficient knowledge of the detailed molecular and cellular mechanisms underlying nephrotic syndrome and thus lack of specific therapeutic targets and treatment guidelines. Steroids remain the standard initial therapy. Depending on the response to steroids, patients fall into steroid-sensitive and steroid-resistant groups. Steroid-resistant patients have a high risk of developing renal failure, while a substantial portion of steroid-sensitive patients experience frequent relapses, develop steroid dependency, and suffer a myriad of serious adverse effects from repeated steroid use. These patients are difficult to treat and face a long battle with RNS.

In order to improve clinical outcomes in patients with RNS, alternative immunosuppressive treatments have been introduced in recent decades, including cytotoxic drugs such as cyclophosphamide, lymphocyte DNA synthesis inhibitors such as mycophenolate mofetil, and calcineurin inhibitors such as cyclosporin and tacrolimus. These agents can be used alone or in combination with steroids. However, the rate of remission induced by different regimens has been highly variable in clinical trials, resulting in a lack of clear consensus regarding the comparative advantages of various combinations. Almost all of the immunosuppressive drugs produce toxic adverse effects that counter their therapeutic benefits. Furthermore, some long-term studies (follow-up for over 10 years) have failed to demonstrate greater remission rates with immunosuppressive therapy than with conservative therapy without immunosuppression [16]. Therefore, the overall risk-benefit profile of immunosuppressive therapy for RNS remains poor.

Considerable progress over the past decade in understanding the molecular mechanisms of nephrotic syndrome has enabled development and testing of novel immunotherapies [17]. Of particular importance is the use of specific B-cell targeting monoclonal antibodies such as rituximab [18]. In several studies, rituximab successfully improved remission rates in patients that had become dependent on other immunosuppressive drugs [18–21]. Based on the hypothesis of circulating permeability factors underlying FSGS and MCD, plasmapheresis has also been attempted in FSGS patients as an alternative approach for treating recurrent nephrotic syndrome following kidney transplantation [22].

Despite the promise that rituximab offers in the treatment of RNS, the available evidence of its efficacy is derived primarily from a few short-term small case studies which showed mixed results [23, 24]. More research is warranted to confirm its efficacy and to observe its long-term safety profile. The prohibitive unit cost of rituximab is also an obstacle to its wide use [25]. Irrespective of different types of conventional therapies and new treatments, a majority of RNS patients still face frequent relapses. Moreover, toxic adverse effects associated with long-term use of immunosuppressive agents continue to present a serious challenge in the clinical management of RNS.

## 3. TCM Views and Treatments for RNS

Because severe edema is the most direct manifestation of RNS, the understanding of RNS in TCM has been based primarily on the theory and clinical experience in treating edema. The pathogenesis of edema was first described in* Yellow Emperor's Inner Classics*, a book written before 100 BC that established the theoretical foundation of TCM [26]. Edema was thought to arise from functional disturbance of three organ systems including Lungs, Spleen, and Kidneys, and the consequent disruption of fluid metabolism. In order to better understand TCM theories, it is important to point out that the definition of organs in TCM is different from that of Western medicine. Organs in the TCM context often constitute functional organ systems with both anatomical and functional meanings. Lungs represent the respiratory system that controls breath and perspiration, Spleen represents the digestive system that controls food assimilation and feces elimination, and Kidneys represent the urinary system that controls urine excretion. A myriad of factors can lead to disorders of these three systems, which separately or collectively result in disruption of fluid homeostasis leading to edema. Therefore, the fundamental TCM treatment strategy for edema is to regulate the functions of Lungs, Spleen, and Kidneys in order to promote fluid elimination through sweat, feces, and urine. When edema is not quickly resolved and becomes chronic, it can block energy flow and blood circulation and thus cause pathological patterns such as Damp Heat, Heat Toxins, Qi Stagnation, and Blood Stasis, leading to secondary complications such as localized or systemic inflammation, ischemia, swelling, pain, stiffness, and skin ulcers. When the root causes of edema and the associated complications are treated accordingly, better therapeutic effects can be achieved [27].

What determines the prognosis, remission, and relapse of nephrotic syndrome? Why are some patients more difficult to treat than others? From the TCM perspective, autoimmune diseases are largely considered to be constitutional diseases. Constitutional characteristics are the result of multiple factors, among which genetics is the decisive factor conferring relative stability and predictability to the constitution. Meanwhile, a person's constitution can be affected by other factors leading to its changeability. This concept is the basis for individuality of disease pathogenesis and responses to treatments. According to the TCM principles first described in* Yellow Emperor's Inner Classics*, personalized treatments must be based on duration of diseases, severity of symptoms, and body constitutions. One of the most important principles is avoiding damaging the patient's Upright Qi, which means a person's internal energy and immunity. In other words, Upright Qi determines the likelihood of remission and relapse of difficult-to-treat diseases. This notion is consistent with the knowledge of disease prognosis in modern medicine, for instance, in cancer treatment [28].

Why is RNS difficult to treat? The reason is its complexity. In the scenario of RNS, symptoms such as severe edema, heavy proteinuria, and hypoalbuminemia have not resolved; serious adverse effects of steroids and other immunosuppressants have persisted; and Upright Qi has been damaged which makes the disease resolution even more difficult. From the perspective of Yin-Yang balance, nephrotic syndrome is mainly characterized as Yang Deficiency and Yin Excess. When nephrotic syndrome becomes RNS, Yang often becomes more deficient, thus worsening the imbalance of Yin-Yang. Other pathological factors such as accumulation of toxins and Blood Stasis (i.e., poor blood circulation) further complicate treatments. Therefore, the crucial TCM concept in treating RNS is to regulate Yin-Yang and Qi-Blood, stabilize the internal environment, and avoid damaging Upright Qi by overtreatment. The comprehensive goal is to maintain biological homeostasis, improve blood circulation and eliminate toxins, minimize the doses and adverse effects of immunosuppressants, improve clinical symptoms, reduce proteinuria, and protect kidney functions in order to improve the overall survival and quality of life of patients with RNS [27].

The TCM strategy for treating edema was first established by Zhang Zhong-Jing (150–219 AD), the most eminent physician in the Chinese history [29]. For readers unfamiliar with Chinese medicine, the significance of Zhang Zhong-Jing and his work can be exemplified by a simple fact that Kampo medicine widely practiced in Japan still uses his original herb formulations with only the slightest changes. The one hundred forty-eight Kampo formulations approved by Japan's national healthcare system are mostly based on his work. These ancient formulations continue to prove valuable for treating modern diseases.

Zhang Zhong-Jing designed many herb formulas to treat edema with varying severities and symptoms (Table 1). Due to the exceptional efficacy of these formulas, they have served as the base formulas for treating edema with subsequent modifications by later generations of physicians. A review of these traditional formulas reveals that* Stephania tetrandra* S. Moore (Fang Ji) and* Astragalus membranaceus *(Huang Qi) are the most important herbs for treating edema.

The clinical effectiveness of* Astragalus membranaceus* in nephrotic syndrome has been consistently demonstrated in many clinical studies [30].* Astragalus *is commonly used in clinical management of proteinuria of various etiologies and is especially effective in glomerulonephritis and nephrotic syndrome [31]. In one study conducted in China, 30 patients with chronic glomerulonephritis, among whom nine were diagnosed with RNS including eight with FSGS and one with IMN, received* Astragalus *therapy [32]. Following 3 weeks of intravenous injections of* Astragalus *extract at 80 g/day, marked reductions in proteinuria were observed in 6 of 8 patients with FSGS, but not in the one patient with IMN. In two separate case reports, patients with RNS due to IMN who had previously failed to respond to immunosuppressive therapy and supportive care were able to achieve complete remission after taking* Astragalus *[33, 34]. For example, a 77-year old patient was treated with supportive therapy (angiotensin-converting enzyme inhibitor, angiotensin receptor blocker, statin, and diuretics) and immunosuppressive agents (cyclosporin and mycophenolate mofetil) for 1 year without any response. After 2 years of unremitting nephrosis, she began oral administration of a Chinese herbal medicine* Nephritis Four-ingredient Pill *(Shen Yan Si Wei Pian) with the active ingredient of* Astragalus *at a dose of 15 g/day. Proteinuria, hypoalbuminemia, hypercholesterolemia, and edema all resolved after approximately 1 year of therapy, and the remission persisted for the next 4 years [33].

Likewise,* Stephania tetrandra* has been widely used for treating nephrotic syndrome and chronic kidney disease, usually in combination with* Astragalus*. In a double-blinded randomized clinical trial (RCT) involving 578 patients with glomerulonephritis in stage 3 chronic kidney disease, we treated patients for 24 weeks with benazepril, a herbal medicine* Stephania*,* and Astragalus Decoction (Fang Ji Huang Qi Tang)*, or the combination of these two [35]. Results demonstrated that the herbal medicine improved renal function shown by significant increases in estimated glomerular filtration rate (eGFR) and hemoglobin with the lowest incidence of side effects; benazepril decreased proteinuria; and the benazepril-herb combination had synergistic effects on reducing proteinuria and protecting kidney functions. Many other studies evaluating* Stephania and Astragalus Decoction* in nephrotic syndrome have reported similar benefits in reducing proteinuria and hyperlipidemia and increasing serum albumin [36–38]. Circulating cytokine levels were affected by the herbal medicine with significant decreases in TNF- and IL-6 and increases in IL-10 [36, 37].

Shenqi particle is a Chinese herbal medicine consisting of 13 herbs including* Astragalus*. Since the 1980s, it has been successfully used to treat various immune-related kidney diseases. Previous small clinical studies showed that Shenqi particle and its components reduced proteinuria in patients with membranous nephropathy [39, 40]. A prospective RCT was conducted in 190 adult patients with membranous nephropathy to compare the efficacy and safety of Shenqi particle with standard therapy of prednisone and cyclophosphamide [41]. After 48 weeks of treatment, both groups showed comparable levels of reduction in proteinuria and similar improvement in serum albumin. However, patients treated with Shenqi particle had significantly higher eGFR compared with baseline, while eGFR was slightly decreased in patients receiving standard therapy. Furthermore, severe adverse events occurred only with standard therapy. These results indicate that Shenqi particle has similar efficacy but fewer side effects compared to standard therapy. It also protects kidney function, while standard therapy does not. Therefore, Shenqi particle is a promising alternative therapy for RNS.

There are more ongoing trials of Chinese herbal medicine in RNS. A double-blinded RCT is currently evaluating the effectiveness of QingReMoShen granules in combination with angiotensin II receptor blocker in the reduction of proteinuria and T lymphocytes in IMN (clinicaltrials.gov, NCT01845688).

## 4. TCM to Support Immunosuppressive Therapy for RNS

As mentioned previously, in TCM theories the basic body constitution for nephrotic syndrome is primarily Yang Deficiency leading to fluid retention with secondary complications such as Blood Stasis. The standard therapy for nephrotic syndrome in conventional medicine is corticosteroids combined with other immunosuppressive drugs. These immunosuppressive therapies often shift the pathology of nephrotic syndrome toward further disequilibrium of Yin-Yang. In the TCM perspective, corticosteroids are pure Yang agents with hot nature. Accumulated fluids in the body, when heated by such Yang agents, inevitably generate Damp Heat leading to Yin Deficiency and Yang Excess. This in turn increases the risk of diabetes, hypertension, coronary heart disease, and osteoporosis, with symptoms such as facial flushing, obesity, acne, insomnia, polydipsia, polyphagia, hyperhidrosis, nocturnal emission, and premature ejaculation. On the other hand, immunosuppressive agents such as cyclophosphamide, mycophenolate mofetil, cyclosporin, tacrolimus, and rituximab are Yin agents with cold nature. Long-term use of these drugs damages Yang Qi and further aggravates Yang Deficiency and Yin Excess, manifested as poor appetite, abdominal bloating, nausea and vomiting, shortness of breath and fatigue, cold intolerance, frequent infection, bone marrow suppression, and reproductive suppression, and so on. Severe Yin-Yang imbalance is therefore the core problem facing patients with RNS, contributing to the chronic recurrent pattern of remissions and relapses as well as serious adverse events.

Based on this notion, the TCM strategy for difficult-to-treat RNS patients who are experiencing serious toxic effects is to restore the Yin-Yang balance and minimize the doses of immunosuppressive drugs in order to reduce adverse effects, improve therapeutic efficacy, and stabilize the internal environment. For patients receiving steroids, herbal formulas that nourish Yin and suppress Yang are used, with* Six-Ingredient Rehmannia Decoction* (Liu Wei Di Huang Tang) as the representative formula in this category. For patients receiving other immunosuppressive agents, herbal formulas that nourish Yang and boost Qi are used, with* Bolster the Spleen Decoction* (Shi Pi Yin) as the representative formula. For patients with low immunity and frequent infections, herbal formulas that support Upright Qi are used, represented by* Eight-Treasure Decoction* (Ba Zhen Tang) and* Jade Windscreen Powder* (Yu Ping Feng San). Depending on the patient's condition, these formulas can be combined and modified to best serve each patient in different phases of disease progression. The commonly used formulas and herb ingredients are listed in Table 2.

*Tripterygium wilfordii *Hook F (TWHF), a traditional Chinese herb with potent anti-inflammatory and immunosuppressive properties, has been used since the 1980s to treat nephrotic syndrome [42]. Several prospective RCTs reported the effectiveness of Tripterygium glycosides (TG), a fat-soluble extract from the root of TWHF, in supporting the use of steroids for IMN treatment [43–45]. When TG monotherapy and TG-steroid combination therapy were evaluated in 84 patients with IMN, following 12 months of treatment, TG alone improved proteinuria, but the combination therapy achieved much higher remission rate than TG alone (76.7% versus 43.9%) [43]. Two subsequent studies compared the efficacy of TG-steroid combination and tacrolimus-steroid combination in IMN [44, 45]. Zuo et al. [44] reported that the two treatments achieved comparable effective rate (76.9% versus 79.9%) at 12 months, but TG-steroid group had lower relapse rate than tacrolimus-steroid group (30.6% versus 52.5%) 6 months after discontinuation of treatment. TG-steroid group also had lower serum creatinine doubling rate, indicating less deterioration of renal functions. A recent meta-analysis of 18 studies analyzing 1,236 adult patients with primary nephrotic syndrome demonstrated greater efficacy of TG combined with steroid than steroid monotherapy [46]. Two earlier meta-analysis reviews also showed beneficial effects of TWHF in inducing remission of nephrotic syndrome and RNS [47, 48].

The chemical constituents of TWHF and its extracts are complex. Among multiple constituents isolated from TWHF, triptolide is a main active ingredient with important anti-inflammatory and immunosuppressive properties [49]. Triptolide can exert anti-inflammatory functions by reducing the release of many proinflammatory cytokines and mediators such as TNF*α*, IL-6, IL-8, and PGE2 [49]. Suppression of T-lymphocyte functions by triptolide is deemed largely responsible for its immunosuppressive properties, ranging from inducing T-cell apoptosis, inhibiting lymphocyte proliferation, regulating CD4^+^ and CD8^+^ cells, and reducing IL-2 and interferon-*γ* production [49–53]. Its immunosuppressive effects have also been attributed to inhibition of dendritic cell maturation and trafficking [54]. In an experimental model of membranous nephropathy, triptolide significantly reduced proteinuria, protected podocytes from C5b-9-mediated injury, and decreased the expression of desmin, a marker of podocyte injury [55].

Great efforts have been dedicated to exploring the potential of integrating Chinese and Western medicine in improving RNS treatment. From 2001 to 2010, more than 150 RCT studies were published that combined Chinese and Western medicine for RNS treatment, most of which were conducted in China and published in Chinese language journals. A meta-analysis selected 11 of the highest quality trials to evaluate the therapeutic effects of combined Chinese herbal medicine and immunosuppressive therapy in the treatment of RNS [56]. The results indicated that patients receiving herbal medicine in combination with immunosuppressive agents had significantly higher complete or partial remission rates and fewer serious adverse events than those receiving immunosuppressive agents alone.

It is important to point out that despite large numbers of TCM clinical trials in this field, high-quality RCT data are limited. Most studies so far were not double-blinded and had small sample sizes (fewer than 100 participants). Many lack details of cointervention and randomization methods. Overall there is a paucity of strong clinical evidence by the standards of modern medicine. In fact, how to design and conduct RCTs to evaluate the efficacy and safety of TCM in accordance with evidence-based medicine is a challenging task facing TCM researchers worldwide (a comprehensive discussion and review of this topic was undertaken by Fung and Linn [57]). Nevertheless, the large body of clinical data and real-world experience available to date has supported the benefits of integrating TCM and conventional medicine for the treatment of RNS.

## 5. Promising Herb-Drug Combinations for RNS

Extensive research in Chinese herbal medicine has identified many active ingredients that have immunosuppressive activity or can improve existing immunosuppressive therapies for treating autoimmune kidney diseases. Of special note are two herbal ingredients and the herb-drug combinations that have been investigated extensively in preclinical and clinical studies. Convincing evidence indicates that they can improve the therapeutic effects and reduce adverse effects of immunosuppressive agents. These two combinations are Wuzhi capsule with tacrolimus and tetrandrine with glucocorticoid.

### 5.1. Wuzhi-Tacrolimus Combination

Of calcineurin inhibitors, tacrolimus is preferred over cyclosporine for treating many types of autoimmune diseases and for preventing graft rejection after organ transplantation. This is largely owing to the fact that tacrolimus has consistently demonstrated better therapeutic effects and fewer adverse effects than cyclosporin in most comparative studies [58–62]. However, narrow therapeutic index and unpredictable bioavailability in humans (5%–67% with average of 27%) have made tacrolimus administration challenging for clinical practice [63]. The high cost of tacrolimus also limits its use.

Wuzhi capsule is an ethanol extract preparation of* Schisandra sphenanthera* (Nan-Wuweizi), a Chinese herb traditionally used for treating hepatitis, liver/kidney deficiency, and neurasthenia. The main active ingredients are schisandrin, schizandrol B, schisantherin A, schisanhenol, and deoxyschizandrin. Owing to its liver-protective, detoxifying, antioxidant, and antitumor activities [64], Wuzhi has been approved for coadministration with tacrolimus for the treatment of drug-induced hepatitis in organ transplant recipients in China [65, 66].

Clinical observations first recognized higher blood levels of tacrolimus when used in combination with Wuzhi. Further studies in humans [67] and animals [68] have confirmed this finding. A meta-analysis [67] evaluating the effects of Wuzhi on tacrolimus pharmacokinetics examined ten RCTs published between 2004 and 2014 that involved 491 patients including mostly kidney or liver transplant recipients and a small number of healthy subjects. Compared with tacrolimus alone, the Tacrolimus-Wuzhi combination significantly increased the plasma concentration of tacrolimus and decreased the dosage of tacrolimus required to maintain the desirable blood concentrations following intervention for one month and three months. These results indicate that Wuzhi capsule can increase the plasma concentration and bioavailability of tacrolimus. The effect appears to be mediated by inhibiting two pathways involved in tacrolimus metabolism, CYP3A and P-glycoprotein, expressed on the intestinal epithelial cells [69, 70].

Based on its successful use in organ transplantation, the use of Tacrolimus-Wuzhi combination was explored for the treatment of autoimmune kidney diseases. In a randomized trial of RNS due to IMN, 60 patients were treated for more than six months with tacrolimus-corticosteroids or tacrolimus-corticosteroids-Wuzhi [71]. The two groups showed similar remission rates, while the group containing Wuzhi used lower doses of tacrolimus and therefore had a higher cost-effectiveness. A recent single-center retrospective study compared tacrolimus alone with the Tacrolimus-Wuzhi combination in 60 patients with autoimmune glomerular disease [72]. The results demonstrated that Wuzhi capsule not only significantly reduced the dosage of tacrolimus required to maintain an effective blood concentration, but also resulted in a higher remission rate (86.7% versus 70.0%) and shorter time to achieve partial remission (2.60 months versus 5.22 months).

All studies to date [65–67, 71, 72] showed that Wuzhi does not increase adverse effects of tacrolimus. In fact, some studies reported improvement in liver functions when Wuzhi was added [67, 72], a finding that is consistent with its known liver-protective activity. Therefore, Wuzhi capsule is a promising tacrolimus-sparing agent for RNS treatment to improve tacrolimus bioavailability, clinical outcome, and pharmacoeconomics.

### 5.2. Tetrandrine-Glucocorticoid Combination

Tetrandrine is another herb ingredient that has received considerable attention with increasing numbers of studies dedicated to understanding its therapeutic effects and biological activities [73]. Tetrandrine is the main active ingredient of* Stephania tetrandra* S. Moore, a key herb known for its effectiveness in reducing edema. Historically it has been used in the treatment of a wide range of inflammatory and autoimmune diseases. Since 1981 it has been approved in China for treating silicosis and rheumatoid arthritis [74]. The purported immunomodulatory activity of tetrandrine has presumably qualified it as a candidate and as an alternative disease-modifying antirheumatic drug (DMARD). Synergistic effects have been observed between tetrandrine and DMARDs such as tacrolimus and cyclosporin in patients with rheumatoid arthritis [75].

Despite abundant clinical evidence showing the benefit of tetrandrine in supporting immunosuppressive treatment of autoimmune diseases, mechanistic studies have been largely lacking. Of note is one earlier report by Seow et al. that demonstrated* in vitro* suppression of mitogen-induced lymphoproliferative responses and antibody production by tetrandrine [76].

Based on our clinical success using herbal formulas containing* Stephania tetrandra *in the treatment of edema and chronic kidney disease [35], we proposed that tetrandrine possesses direct immunosuppressive activity or can potentiate the effect of corticosteroids. We therefore carried out studies to investigate the potential synergistic interaction between tetrandrine and methylprednisolone and the possible mechanisms of action.

Mitogen-activated human peripheral blood mononuclear cells (PBMCs) are preferred over isolated T cells as an* in vitro* model for studying the human immune network. We first tested the immunosuppressive effect of methylprednisolone combined with tetrandrine in PBMCs from healthy human subjects [77]. Tetrandrine significantly decreased the half maximal inhibitory concentration (IC_50_) value of methylprednisolone even at the lowest concentration of 0.3 nM, and no toxic effect was observed with tetrandrine even at the high concentration of 300 nM. Tetrandrine and methylprednisolone both suppressed the production of proinflammatory cytokines TNF-*α* and IL-6, and the combination showed stronger inhibitory effect. Tetrandrine appears to potentiate the immunosuppressive activity of methylprednisolone via at least two mechanisms. First, it inhibits the function of drug efflux pump P-glycoprotein 170 in T cells and thus increased the intracellular methylprednisolone concentration. Second, tetrandrine in combination with methylprednisolone synergistically inhibits the phosphorylation of mitogen-activated protein kinase family, specifically ERK1/2. However, CD4^+^ CD25^+^ Foxp3^+^ regulatory T cells targeted by some other immunosuppressive drugs such as methotrexate are not affected by tetrandrine and methylprednisolone.

Subsequently, we evaluated the immunosuppressive pharmacodynamics of tetrandrine alone and in combination with methylprednisolone in PBMCs of hemodialysis patients [78]. Tetrandrine alone inhibited the proliferation of PBMCs, with a median (range) IC_50_ value of 1.61 (1.04–4.79) *μ*M. At lower concentrations (0.3–300 nM), tetrandrine significantly decreased the IC_50_ value of methylprednisolone. According to a study on the pharmacokinetics of tetrandrine, the maximum blood concentration of tetrandrine after ingesting 40 mg tetrandrine could reach 17 *μ*M in healthy volunteers [79]. Thus tetrandrine can augment the immunosuppressive effect of methylprednisolone at clinically relevant doses without risks of side effects.

Collectively, our results demonstrate that tetrandrine is not only an immune inhibitor by itself, but also synergistically potentiates the immunosuppressive efficacy of glucocorticoids in healthy subjects and hemodialysis patients. This finding provides a mechanistic explanation to the observed clinical benefits of* Stephania tetrandra* in autoimmune diseases. It also offers a clear rationale for using the combination of tetrandrine and glucocorticoids to reduce steroid resistance and attenuate toxic side effects of steroids.

## 6. Limitations and Future Studies

It is worth noting that some Chinese herbs mentioned above are associated with potential side effects.* Stephania tetrandra* S. Moore (Fang Ji) is a safe herb. However, historically* Aristolochia fangchi* (Guang Fang Ji) was sometimes mistakenly used as* Stephania tetrandra* S. Moore, partly due to their similar Chinese names. This contributed to aristolochic acid nephropathy events first discovered in Belgium in 1993 [80] and later found widely in China and other Asian regions [81]. Although most countries have banned* Aristolochia fangchi*, we should remain cautious because herbs containing aristolochic acid are still used in traditional herbal remedies [82].* Tripterygium wilfordii* Hook F. and its extract triptolide, as potent immunosuppressive agents, share features common to immunosuppressants including toxicity. Short-term administration of the low doses traditionally used in TCM practice does not appear to have toxic effects. However, long-term high-dose treatments can cause liver dysfunction, leukopenia, and damage of the reproductive system [83]. Moreover, TCM emphasizes that herb medicine be prescribed based on the comprehensive differential diagnosis of a patient's conditions guided by TCM theories. Otherwise, if herbs are used inappropriately, even nontoxic herbs can cause adverse effects. Therefore, to avoid and minimize side effects, Chinese herbal prescription should follow the diagnosis and treatment principles of TCM.

Current research in this field is limited in several aspects. First, from the perspective of evidence-based medicine, many clinical trials have been poorly designed and have yet to provide robust evidence of efficacy. Therefore, more high-quality clinical studies with large sample sizes are needed to validate the efficacy and safety of Chinese medicine for RNS treatment. A prevailing methodologic drawback in most clinical trials in TCM has been the exclusion of TCM principles in the study design [57]. When Chinese herbal medicine is used to treat a Western medical diagnosis without following TCM principles, any lack of efficacy can arguably result from the mismatch of treatments and diseases in the TCM framework. Therefore, study designs incorporating TCM theories are necessary to improve future RCTs in TCM. Second, there is a lack of understanding of the molecular mechanisms for herbs that have shown promising results in treating RNS. This applies to multiherb formulations, for example,* Stephania and Astragalus Decoction*, and purified components from single herbs, for example, tetrandrine from* Stephania tetrandra* S. Moore. Not only is detailed and in-depth basic research important to elucidate the mechanisms underlying the therapeutic effects of herbal medicine, but it also has the potential to reveal novel signaling pathways and treatment targets for RNS. Finally, investigations of empirical herbal prescriptions that have been well-tested in clinical practice will likely lead to identification of effective herbs and active compounds not previously recognized.

## 7. Summary

Recent advances in understanding the pathogenesis of RNS have led to development and testing of new immunosuppressive agents. Although this has resulted in higher remission rates in some RNS patients, poor clinical outcomes persist due to its chronic relapsing nature as well as unsatisfactory efficacy and toxic adverse effects of available immunosuppressive regimens. The urgent need for alternative treatments has prompted renewed interests in exploring traditional Chinese medicine for safer and more effective therapies for RNS. These efforts have provided evidence supporting the integration of Chinese herbal medicine and conventional therapies. Chinese herbal medicine can improve clinical symptoms, reduce proteinuria, and protect kidney functions of RNS patients through regulation of the internal environment. Some herbs can modulate immune function by affecting immune cells directly and thus can be used as alternative treatments when existing therapies have failed. Other herbs can be coadministered with steroids and other immunosuppressants and have synergistic effects in improving therapeutic efficacy and decreasing adverse events of existing therapies. Current research in this field has been limited by relatively low quality of clinical trials and lack of mechanistic studies. In the era of evidence-based precision medicine, more high-quality clinical studies with large sample sizes and incorporation of TCM principles in the study design are necessary to validate the efficacy and safety of Chinese medicine. More basic research is needed to elucidate the mechanisms of action underlying the therapeutic effects of herbal medicine for treating RNS. The great potential that traditional Chinese medicine holds for RNS warrants such efforts.

## Figures and Tables

**Table 1:** Traditional herb formulas and ingredients for treating edema.

Indications	Formulas	Herbs
Mild edema	*Stephania & Astragalus Decoction* (Fang Ji Huang Qi Tang)	*Stephania tetrandra* (Fang Ji), *Astragalus membranaceus* (Huang Qi), *Atractylodes macrocephala* (Bai Zhu), *Glycyrrhiza uralensis* (Gan Cao)

Moderate edema	*Stephania & Poria decoction* (Fang Ji Fu Ling Tang)	*Stephania tetrandra* (Fang Ji), *Astragalus membranaceus* (Huang Qi), *Glycyrrhiza uralensis* (Gan Cao), *Poria cocus* (Fu Ling), *Cinnamomum cassia* (Gui Zhi)

Severe edema	*Cocculus decoction* (Mu Fang Ji Tang)	*Cocculus orbiculatus *(Mu Fang Ji), *Panax ginseng* (Ren Shen), *Cinnamomum cassia* (Gui Zhi), *Gypsum* (Shi Gao)

Recurrent edema	*Cocculus minus Gypsum plus Poria plus Mirabilite decoction* (Mu Fang Ji Qu Shi Gao Jia Fu Ling Mang Xiao Tang)	*Stephania tetrandra* (Fang Ji), *Panax ginseng* (Ren Shen), *Cinnamomum cassia* (Gui Zhi), *Mirabilite* (Mang Xiao), *Poria cocos *(Fu Ling)

Severe edema with constipation and urinary retention	*Stephania Zanthoxylum Descurainia Rhubarb Pill* (Ji Jiao Li Huang Wan)	*Stephania tetrandra* (Fang Ji), *Zanthoxylum bungeanum *(Jiao Mu),* Descurainia sophia* (Ting Li Zi), *Rheum officinale *(Da Huang)

**Table 2:** Herb formulas and ingredients commonly used for reducing side effects of immunosuppressive agents.

Indications	Formulas	Herbs
Side effects of steroids	*Six-Ingredient Rehmannia Decoction *(Liu Wei Di Huang Tang) & derivatives	*Rehmannia glutinosa* (Sheng Di Huang), *Dioscorea opposita* (Shan Yao), *Cornus officinalis *(Shan Zhu Yu), *Poria cocos *(Fu Ling), *Alisma orientale* (Ze Xie), *Paeonia suffruticosa *(Mu Dan Pi), *Glehnia littoralis* (Bei Sha Shen), *Ophiopogon japonicus* (Mai Men Dong), *Paeonia lactiflora* (Bai Shao), *Lycium barbarum *(Gou Qi Zi), *Anemarrhena asphodeloides* (Zhi Mu)

Side effects of other immunosuppressants	*Bolster the Spleen Decoction* (Shi Pi Yin) *Kidney Qi Pill* (Jin Gui Shen Qi Wan) & derivatives	*Aconitum carmichaelii* (Fu Zi), *Epimedium* (Yin Yang Huo), *Morinda officinalis *(Ba Ji Tian), *Rehmannia glutinosa* (Shu Di Huang), *Atractylodes Macrocephala *(Bai Zhu), *Codonopsis pilosula* (Dang Shen), *Astragalus membranaceus* (Huang Qi), *Zingiber officinale* (Gan Jiang), *Cinnamomum cassia* (Gui Zhi), *Alpinia Katsumadai *(Cao Dou Kou), *Amomum tsao-ko *(Cao Guo), *Cuscuta australis *(Tu Si Zi), *Cistanche deserticola* (Rou Cong Rong)

Hypoimmunity and frequent infections	*Eight-Treasure Decoction* (Ba Zhen Tang) *Jade Windscreen Powder* (Yu Ping Feng San)	*Astragalus membranaceus* (Huang Qi), *Panax ginseng *(Ren Shen), *Atractylodes Macrocephala* (Bai Zhu), *Glycyrrhiza uralensis* (Gan Cao), *Angelica sinensis *(Dang Gui), *Cordycepssinensis *(Dong Chong Xia Cao), *Ganoderma lucidum *(Ling Zhi)
